# RURAL AGE-FRIENDLY ECOSYSTEMS: A SCOPING REVIEW WITH RECOMMENDATIONS FOR AGE-FRIENDLY COMMUNITIES

**DOI:** 10.1093/geroni/igac059.3010

**Published:** 2022-12-20

**Authors:** Daniel Liebzeit, Anna Krupp, Jacinda Bunch, Harleah Buck

**Affiliations:** University of Iowa, North Liberty, Iowa, United States; University of Iowa, Iowa City, Iowa, United States; University of Iowa, Iowa City, Iowa, United States; Csomay Center for Gerontological Excellence, Iowa City, Iowa, United States

## Abstract

The population of older adults in rural areas is rising, and they experience higher rates of poverty and chronic illness, have poorer health behaviors, and experience different challenges than those in metropolitan areas. Promoting age-friendly rural communities and healthcare is key to improving outcomes and addressing disparities. This international scoping review seeks to 1) map the state of the science of age-friendly systems in rural areas regarding structural characteristics, processes for delivering age-friendly practices, and outcomes of age-friendly systems, 2) analyze strengths, weakness, opportunities, and threats of age-friendly system implementation, and 3) make person, practice, and policy-level recommendations to support active aging. Articles were retrieved from PubMed, CINAHL, AgeLine, PsychINFO, EMBASE, Scopus, and Academic Search Elite on 10/26/21 and included if they used age-friendly framing, self-identified as rural, and reported empiric data. Data were charted across three analytic layers: socioecological model, Donabedian’s framework, and SWOT analysis. While age-friendly systems in this review were heterogeneous, many utilized the World Health Organization age-friendly cities framework. Results reveal limited data on outcomes relevant to organizations. While the SWOT analysis revealed many strengths of age-friendly systems, it also revealed several weaknesses, threats, and gaps. Weaknesses included over-reliance on trained volunteers and staff, communication, and teamwork. Threats included community and health system barriers and challenges in developing regions. Despite the benefits of age-friendly systems, we must acknowledge limitations of the evidence base, pursue opportunities to examine organizational metrics to support implementation and sustainability of age-friendly systems, and leverage improvements in age-friendliness at a community level.

